# Headaches and obesity

**DOI:** 10.1590/0004-282X-ANP-2022-S106

**Published:** 2022-08-12

**Authors:** Ida Fortini, Bernardo Dror Felsenfeld

**Affiliations:** 1 Universidade de São Paulo, Faculdade de Medicina, Hospital das Clínicas, Departamento de Neurologia, São Paulo SP, Brazil.

**Keywords:** Body Mass Index, Obesity, Headaches, Migraine without Aura, Pseudotumor Cerebri, Índice de Massa Corporal, Obesidade, Cefaleia, Enxaqueca sem Aura, Pseudotumor Cerebral

## Abstract

Obesity and headache disorders are two very common conditions in the general population that have been increasing in incidence over the last decades. Recent studies have shown a significant relationship between obesity and headaches, particularly migraine, with an important role in whether the disease is chronic. On the other hand, no such association was found with tension-type headaches. Studies showing an overlapping of hunger-control pathways and those involved in the pathophysiology of migraine may justify the close association between obesity and migraine. Moreover, a secondary headache for which obesity is a strong risk factor is idiopathic Intracranial Hypertension (pseudotumor cerebri), with several studies showing the impact of weight reduction/bariatric surgery in the treatment of the disease. In conclusion, since obesity is a modifiable risk factor, it is important for physicians treating patients with headaches, and particularly migraine, to be aware of the association between these two disorders.

## RESUMO

Obesidade e cefaleias são duas condições muito frequentes na população geral e que vem aumentando em incidência nas últimas décadas. Estudos recentes têm demonstrado uma significativa relação entre obesidade e cefaleia, particularmente na enxaqueca, com papel importante na cronificação da doença. Por outro lado, não se observa tal associação com cefaleia do tipo tensão. Trabalhos que mostram uma sobreposição das vias de controle da fome e daquelas envolvidas na fisiopatologia da enxaqueca podem justificar a íntima associação entre obesidade e enxaqueca. Além disso, uma cefaleia secundária para a qual a obesidade é um forte fator de risco é a Hipertensão Intracraniana Idiopática (pseudotumor cerebral), inclusive com diversos estudos mostrando o impacto da redução do peso/cirurgia bariátrica no tratamento da doença. Concluindo, visto que a obesidade é um fator de risco modificável, é importante para os médicos que tratam de pacientes com cefaleia e, particularmente, enxaqueca, estarem cientes da associação entre essas duas comorbidades.

## INTRODUCTION

Obesity and headaches in general, and migraine in particular, are frequent conditions in the general population, and the complaint of a headache is one of the most frequent reasons for consultation in neurology clinics. Migraine is the leading cause of disability in people under 50 years of age worldwide, generating great impairment of quality of life^1^.

The prevalence of overweight and obesity has increased substantially in recent decades, and obesity is one of the main risk factors for diseases of various kinds and death worldwide^2^ ^,^ ^3^.

According to WHO criteria, obesity is defined by the percentage of total body fat, which is 35% or more for women and 25% or more for men^4^. However, given the difficulties in measuring total body fat, most epidemiological studies use anthropometric indices such as body mass index (BMI) to estimate total body obesity (TBO) and abdominal circumference (AC) to estimate abdominal obesity (AO). Based on BMI, obesity is considered when BMI ≥30 kg/m^2^ and, based on AC, AO when AC is greater than 88 cm in women and 102 cm in men^5^ ^,^ ^6^.

In the US, the prevalence of overall obesity (BMI ≥30) increased from 33% (women) and 27% (men) in 1999-2000 to 35% (women) and 32% (men) in 2007-2008. Also in the US, the prevalence of AO has increased over the past decade, with 62% of women and 43% of men meeting criteria for AO in 2007-2008, compared to 56% of women and 38% of men in 1999-2000^6^ ^,^ ^7^. It must be taken into account that there are racial differences in the distribution of adipose tissue, and data show that the prevalence of obesity in the US is higher in African-Americans than in Caucasians, and that it is higher than in people of Asian descent^8^. 

According to 2017 data from the Brazilian Ministry of Health, 52.6% of men over the age of 18 are overweight, as are 44.7% of women in the country, and this proportion increases with increasing age, reaching 63% of men between the ages of 34 and 65, and 55.9% of women between the ages of 45 and 54^9^.

Obesity carries a substantial personal and financial burden, and is comorbid with several clinical conditions, such as insulin resistance, type 2 diabetes, hypertension, dyslipidemia, heart disease, cancer, mood and sleep disorders, reproductive problems, liver disease, and pain syndromes. 

On the other hand, there is controversy as to whether the incidence and prevalence of migraine has also increased in recent decades. Many studies have reported that the incidence and/or prevalence of migraine in adolescents and adults has increased, especially from the late 1980s to the late 1990s and from the mid to late 1990s to the early 2000s^8^. However, in the study by Lynberg et al., which assessed changes in migraine prevalence over time, no increase in migraine prevalence was observed between 1989 and 2001^10^. Nevertheless, the study methodologies limit the ability to draw definitive conclusions about this, and further studies using ICHD-3 criteria should be conducted to clarify this issue. 

## RELATIONSHIP BETWEEN HEADACHES IN GENERAL AND OBESITY

Brown et al. conducted the first population-based cross-sectional study looking at the association between obesity and headaches of any type by studying 14,779 young women (18 to 23 years old) compared to controls (young women who did not report headaches). They found that women with BMI ≥30 were 47% more likely to report migraine or headaches than non-obese women (OR 1.47, 95% CI 1.25-1.73)^11^. 

In a first prospective longitudinal study by Scher et al. in the USA 1,192 adults (aged 18-65) with episodic headaches [EH] (2-104 headache days/year) or chronic daily headache [CDH] (≥180 headache days/year) assessed at baseline and after 11 months were evaluated. The authors found that self-reported obesity at study start (BMI ≥30) was 34% more common in individuals with CDH (OR 1.34, 95% CI 1.0-1.8) than with EH. At assessment at 11 months participants with EH with obesity were five times more likely to have progressed to CDH than those who were non-obese with EH (OR 5.28, 95% CI 1.3- 21.1)^12^. 

Keith et al. conducted a cross-sectional analysis of over 220,000 female participants aged 16 to 90 and found that the risk of headache in general was higher in obese people and that the risk of headaches was higher with an increasing degree of obesity . Participants with BMI ≥ 30 had about 35% higher risk of headache than participants with normal BMI. For subjects with morbid obesity (BMI ≥ 40), the risk of headaches increased by about 80%^13^. 

## RELATIONSHIP BETWEEN MIGRAINE AND OBESITY

Population studies have consistently identified an association between obesity and headaches, and particularly with migraine^8^ ^,^ ^11^ ^,^ ^12^ ^,^ ^14^ ^-^ ^16^. 

A population-based study of 5,847 adolescents (aged 13-18) by Robberstad et al. found that those with headaches or migraine were 60% more likely to be overweight or obese (based on BMI) than those without migraine (OR 1.6, 95% CI 1.4-2.2)^17^. 

Another cross-sectional study of 3,733 women of childbearing age also confirmed the obesity-migraine relationship. The study also established that migraine risk increased with increasing obesity. The overall odds of migraine in women with obesity (BMI 30-34.9) increased almost 1.5 times compared to non-obese women (OR 1.48, 95% CI 1.12-1.96). Women with grade II obesity (BMI 35-39.9) had a two-fold increased risk of migraine (OR 2.07, 95% CI 1.27-3.39) and for women with morbid obesity (grade III; BMI ≥40) the OR was 2.75, 95% CI 1.60-4.70 of having migraine^18^. 

Peterlin et al. examined the association of episodic migraine (EM) and obesity and the influence of age, race, and gender in 3,862 adult participants. They reported that obese individuals had an 81% increased risk of EM compared to those of normal weight (OR 1.81, 95% CI 1.27-2.57; P = 0.001). Subgroup analyses demonstrated that the odds of low frequency EM [LF] (≤108 headache days/year) and very low frequency EM [VLF] (≤60 headache days/year) increased by 83-89% in those with obesity (EM LF: OR 1.83, 95% CI 1.26-2.65; EMVLF: OR 1.89, 95% CI 1.29-2.78) compared to those with normal weight. No significant increase in mean headache frequency were observed in participants with EM based on the degree of obesity from normal, to overweight to obese. This study also provided support for the influence of age on the obesity-migraine relationship. The results stratified by age showed that the risk of EM in the obese was increased by 86% in participants < 50 years of age (OR 1.86, 95% CI 1.20-2.89; p=0.006), but was not significantly increased in those > 50 (OR 1.15, 95% CI 0.61-2.18)^19^.

A Chinese study evaluated 5,049 male and female participants, aged 18 to 65 years. Migraine was defined according to the ICHD criteria. No association was identified between migraine and those with BMI < 30. Compared to those with normal BMI (18.5-23), those with BMI ≥ 30 had a higher prevalence of migraine (8.6 v. 13.8 %, p = 0.001). Multivariate adjusted *odds ratio* demonstrated that those with morbid obesity were more than twice as likely to have migraine [OR 2.10 (1.39-3.12)] compared to those with BMI between 18.5 and 23. However, no association was observed between obesity and migraine severity, frequency, or disability^20^. 

A meta-analysis by Gelaye et al., 2017, covering 288,981 participants from 12 different studies found that the age and sex-adjusted combined risk of migraine in obese patients increases by 27% compared to those of normal weight (OR = 1.27; 95% CI: 1.16 -1.37, p < 0.001)^21^.

Most population-based studies of obesity and migraine were based on BMI rather than AO. However, AO seems to be a better measure of obesity than BMI^16^. Abdominal visceral fat is metabolically different from other body fats, and appears to be an independent risk factor for clinical complications. It should be considered that both fat distribution and migraine prevalence vary substantially with gender and age, and it is possible that the relationship between the two also changes with these factors^14^ ^,^ ^19^ ^,^ ^22^ ^,^ ^23^. 

TBO, as measured by BMI, has been associated with both increased migraine prevalence and progression from episodic to CM^12^ ^,^ ^14^ ^,^ ^16^ ^,^ ^19^ ^,^ ^21^ ^,^ ^22^ ^,^ ^24^ ^,^ ^25^. 

A recent large population-based study by Kristoffersen et al. that used data from the HUNT3 study (*third population-based Nord-Trøndelag Health Study*), examined the relationship between obesity and headaches. They took into account body fat distribution, migraine subtypes, and TBO^16^. Both TBO and AO were associated with a higher prevalence of migraine when compared to controls without headache (OR 1.45 95% CI 1.32-1.59 and OR 1.29 95% CI 1.18-1.41, respectively), particularly for individuals < 50 years of age (OR 1.74 95% CI 1.54-1.98 and OR 1.89 95% CI 1.69-2.11). Similar results were obtained for migraine with and without aura. Overall, weaker associations were seen between obesity and CTT. In addition, some migraine features are affected in the overweight population and a dose-response relationship was found between obesity levels and increased headache frequency in individuals with migraine. TBO was associated with migraine prevalence and attack frequency independent of AO. Regarding body fat distribution, the association between migraine and TBO was independent of AO, but not vice versa, suggesting that TBO may be a more important measure regarding migraine prevalence and whether the migraine is considered chronic^16^. 

## OBESITY AND CHRONIC MIGRAINE (CI)

In a cross-sectional analysis of 30,849 participants by Bigal et al., the probability of chronic migraine (CM) in patients with obesity *v.* controls (obese patients without headaches or with ≤108 headaches/year) *v.* those with BMI of 18.5-24.9 was increased by 50% in individuals with BMI between 30 and 34.9 (OR 1.5, 95% CI 1.2-1.8) and by 100% in those with BMI > 35 (OR 2.0, 95% CI 1.4-2.4)^24^.

According to Diener and Beck, individuals with episodic headaches and obesity are five times more likely to develop chronic daily headaches (CDH) than normal weight individuals^26^.

A cross-sectional study of the general population by Schramm et al. of 9,685 participants diagnosed with migraine based on the ICHD-2 showed that participants with CM were 72% more likely to be obese (BMI > 30) than those without headaches (OR 1.72, 95% CI 1.02-2.92). However, this finding was no longer significant after adjusting for analgesic use (OR 1.85, 95% CI 0.54-6.27)^27^. Nevertheless, a link between the two diseases is generally recognized. A study reporting improvement in migraine after marked weight loss reinforces the hypothesis that both diseases have an impact on each other^28^. 

## OBESITY AND TENSION-TYPE HEADACHES

Few data are available regarding the association of tension type headache (TTH) and obesity, but three population-based studies have evaluated this association. Robberstad et al. reported a 40% increased risk of episodic TTH (ETTH) and chronic TTH (CTTH) headaches in overweight or obese adolescents aged 13 to 18 (OR 1.4, 95% CI 1.1-1.6)^17^. 

Bigal et al. found no association between ETTH and obesity in 2,051 adult subjects with ETTH^24^. A 40% increased risk of CTTH was found only in those with BMI ≥35 compared to those with BMI of 18.5-24.9 (OR 1.4, 95% CI 1.1-1.9) However, after adjusting for other covariates, (gender, age, race, use of headache medications, sleep problems, educational level, and depression), BMI was not associated with CTTH diagnosis^24^. 

Table 1 shows data from some studies on the relationship of obesity and headaches. 

**Table 1.** Some headache and obesity studies.

Authors/ year of publication	n	Population included	Gender	Median age (range)	BMI	Primary outcome	Results
Brown et al. 2000^11^	12.855	Headache/ Mig X Controls without headache	F	20 (18-23)	BMI < 17 BMI 18-24.9 BMI 25-24.9 BMI 25-29.9 BMI 30-34.9 BMI: 35-39.9 BMI > 40	OR headache or Mig	OR headache or Mig in women with BMI 25-30: 1.12 BMI >30 (OR 1.47) X normal BMI
Scher et al. 2003^12^	1932	Episodic headache X CDH	F and M	40 (18-65)	BMI < 25 BMI 25-30 BMI ≥30	Prevalence of headache OR X CDH X incidence of CDH OR in patients with prior EM	OR CDH 5 X > in EM patients with BMI ≥ 40 (OR 5.28)
Bigal et al. 2006^25^	30.215	No headache, <14 headache days (non Mig), CDH, EM X Controls	F and M	30 (18-89)	BMI <18.5 - n = 941 BMI 18.5- 24.9*†* (n = 15,501) BMI 25-29.9 (n = 9,258) BMI 30-34.9 (n = 3,133) BMI ≥35 (n= 1,382	OR of EM X No Mig, No headache or CDH	BMI 25-29.9 (OR 1.4, IC 95% 1,1 -1,8) X normal BMI BMI ≥ 30: (OR 1,3, IC 95% 1,1-1,6) IMC ≥ 40 (OR 1,8 IC 95% 1,4-2,2)
Ford et al. 2008^17^	7601	Severe headache or Mig X No severe Mig or severe headache	F and M	46 (20-85)	BMI <18.5 - (n = 121) BMI 18.5-24.9*†* (n = 2313) BMI 25-29.9 (n = 2784) BMI ≥30 (n= 2383)	OR Mig	OR 1,37 of Mig or severe headache in patients with BMI ≥ 30 X normal BMI
Peterlin et al. 2010 ^14^	15.631	Severe Mig or headache X no severe headache or severe Mig	F and M	38 (20-55)	1) BMI ≥30 (n = 4,585) BMI <30*†* (n = 11,046) 2) AO (n = 6,631) No AO (n = 9,000) AO: AC ≥88 cm for women and AC ≥102 cm for men	OR Mig	OR Mig or severe headache with BMI ≥ 30 X BMI nl Women OR: 1.39. Men OR: 1.39
Robberstad et al. 2010 ^17^	5847	Mig, TTH, headache without classification X Controls with no Mig or headache	F and M	<18 (13-18)	Weigh excess (based on BMI pediatric by sex and age) - n = 891	OR Mig	OR Mig > adolescents with BMI > 25
Vo et al. 2011^18^	3733	Mig X Controls	F	<40 (18-40)	BMI < 18.5 (n = 160) BMI 18.5-24.9*†* (n = 2623) BMI 25-29.9 (n =612) BMI ≥ 30 (n= 338)	OR EM	OR 1.81 of EM in patients with BMI ≥ 30 X normal BMI
^20^	5029	EM X Controls CDH excluded	F and M	43 (18-46)	BMI < 18.5 (n = 262) BMI 18.5-<23*†* (n =2,469) BMI 23-<25 (n =1,064) BMI 25-<30 (n = 1,044) BMI ≥ 30 (n = 190)	OR EM	OR EM 2.10 in patients with BMI ≥ 30 X normal BMI
Peterlin et al. 2013^19^	3862	EM X no headache	F and M	< 46.6 (18-50+)	BMI < 18.5 (n = 129) BMI 18.5- 24.9*†* (n =1424) BMI 25-29.9 (n =1306) BMI ≥ 30 (n= 1004)	OR EM	OR EM 1.81 in patients with BMI ≥ 30 X normal BMI
Kristoffersen er at. 2020^16^	33.176	Mig, TTH X Controls	F and M	No headache 56.7 (SD 15.6) Mig 46.2 (SD 12.9) TTH 49.3 (SD14.0)	BMI < 18.5 BMI 18.5 - 24.9 BMI 25 - 29.9 BMI ≥ 30 Waist circumference (AO)	OR Mig OR TTH	TBO and AO: associated with a higher prevalence of Mig X headache-free controls (OR 1.45) and OR 1.29 95% respectively), particularly for individuals < 50 years of age (OR 1.74 and OR 1.89). Similar results in MO and MA. Weaker associations were observed between obesity and TTH. TBO associated with Mig prevalence and attack frequency independent of AO.

N: number of participants; BMI: body mass index; F: female; M: male; Mig: migraine; EM: episodic migraine; X: versus; CDH: chronic daily headache; TTH: tension type headache; MO: migraine without aura; MA: migraine with aura; AO: abdominal obesity; CA: abdominal circumference; TBO: total body obesity.

## OBESITY AND IDIOPATHIC INTRACRANIAL HYPERTENSION (IIH)

IIH (pseudotumor cerebri) is characterized by a progressive headache associated with increased intracranial pressure (ICP) > 25 cm H2O, normal cerebrospinal fluid (CSF) chemistry, and absence of other structural, vascular, metabolic, toxic, or hormonal causes of increased ICP. Patients often present with increased blind spot, papilledema, visual field deficits, and occasionally sixth nerve palsy. Headaches are reported by more than about 80% of patients with ICH and are often the first symptom reported^29^. 

IIH is much more common in women than in men (about 84-90% of women in the studies). The association between obesity and IIH is much more robust in women, and in case-control studies, while 25-65% of men with IIH are overweight, this percentage is about 80% in women with ICH. However, even in men with IIH, the prevalence of obesity is higher than in healthy male controls^8^. 

The average age-adjusted annual incidence rate for IIH is about 1-3 per 100,000 in the general female population. In women with obesity the incidence is 12-19 per 100,000. Regarding the prevalence of IIH, this is about 11 per 100,000 in the general female population and 86 per 100,000 in obese women^29^ ^,^ ^30^. 

Small case-control studies have shown that both BMI and weight gain are risk factors for IIH^8^ ^,^ ^30^. 

Although IIH can occur in people of normal weight, both high BMIs and larger percentage weight gains are associated with a progressively higher risk of IIH. According to Hamdallah et al. a one-year weight gain of 5-10% is associated with an OR 3.6 (95% CI 1.1-11.9, P = 0.04) of IIH; 11-15% to an OR 10.2 (95% CI 1.9-56.5, P = 0.008) and of more than 15% to an OR of 15.2 (95% CI 1.5-151.2, P = 0.02)^31^. 

A study by Kesler et al. found that lower body adiposity (defined as a weight-hip ratio < 0.76) was more than six times more common in patients with IIH than in the control group of obese women^32^.

## CENTRAL REGULATION OF FEEDING - OVERLAP WITH MIGRAINE PATHOPHYSIOLOGY

Feeding regulation is carried out by several hypothalamic nuclei and their connections. The arcuate nucleus is a major center of feeding regulation, where orexinergic neurons containing NPY and agouti-related protein (AgRP) and neurons containing anorexinergic neuropeptides, such as proopiomelanocortin and CART (cocaine and amphetamine-regulated transcript), are found. 

The sympathetic nervous system and the release of adipokines, such as leptin and adiponectin, are involved in peripheral mechanisms of feeding control. 

Signals coming from the gastrointestinal (GI) tract related to nutrients and hormones such as leptin, insulin, glucose, peptide YY3-36, ghrelin, and CCK repress orexinergic neuropeptides and stimulate anorexinergic neuropeptides. Therefore, an imbalance in the regulation of metabolism or the anatomical structures involved could trigger obesity^33^ ^-^ ^35^. 

As demonstrated by functional magnetic resonance imaging studies, the hypothalamus has a primary role in the pathophysiology of migraine. The premonitory symptoms of the crises (yawning, drowsiness, and mood, sleep, and appetite changes) have hypothalamic origin. Therefore, it is hypothesized that pathological modulation of the hypothalamus in migraineurs may result in hyperphagia and weight gain. Several hypothalamic peptides, proteins, and neurotransmitters involved in eating contribute to the pathophysiology of migraine (serotonin, orexin, and adipokines). It is also possible that modulation of these hypothalamic peptides and proteins in association with the urge to eat or not to eat and/or by states of obesity may trigger or contribute to the generation of migraine attacks^36^. 

Although several hypotheses have been postulated in recent years, the association between migraine and obesity remains unclear. A number of lifestyle factors may be important confounders that could explain the possible relationship between migraine and obesity, such as smoking, physical activity level, altitude, blood pressure, stress, sleep alterations, and medication use. However, a relationship between migraine and obesity is conceivable because several common biochemical markers and the influence of central and peripheral mechanisms are involved in the pathophysiology of obesity and migraine^14^ ^,^ ^17^ ^,^ ^22^.

Of the various biomarkers that may be important in the relationship between obesity and migraine, and also with insulin resistance (IR) and diabetes, we highlight a few below. 

## Serotonin

Interictal plasma serotonin levels are low in migraine patients and increase transiently during attacks. Studies in animal models show that blockade of the 5-HT2C receptor is associated with increased eating and development of obesity. Low interictal serotonin states in migraine patients lead to increased urge to eat. Elevated levels in the ictal period may promote hypophagia^34^. Several drugs that modulate serotonin and its receptors, including the receptors most directly implicated in satiety 5-HT1B and 5-HT2C are used in the treatment of migraine^35^. 

### Adipocytokines

Adipokines (ADP) participate in energy homeostasis, immunity and inflammation. They are proteins secreted mainly by adipocytes and their receptors and are found throughout the vascular endothelium and in the brain, including the hypothalamus. Women have higher levels of ADP than men. Most studies show that ADP levels are inversely associated with obesity (obese people have lower fasting ADP levels)^23^ ^,^ ^35^ ^,^ ^36^.

### Orexin

Associated with headache and satiety, orexin A (OXA) and orexin B are peptides from hypothalamic neurons with axonal projections to the cortex and brainstem, superficial lamina of the spinal cord, and GI tract^37^. Animal and human data demonstrate the role of orexins in pain processing. Administration of intrathecal OXA in rats has antihyperalgesic properties^37^. The role of orexins and their receptors in migraine is still being studied, but OXA levels are shown to be elevated in the CSF of patients with CDH. These findings may suggest orexin resistance or orexin receptor blockade in patients with CDH (which explains the ongoing pain with elevated OXA levels)^35^. 

### Leptin

Produced mainly by adipocytes, but also by various other tissues, including in the brain, leptin is inhibited by testosterone and increased by ovarian steroids. Women exhibit 2-3 times higher levels than men, even when matched for age and BMI^14^. Leptin is associated with satiety, but obese people generally exhibit high circulating concentrations, suggesting a state of leptin resistance in obese states. It has also been implicated in modulating inflammation and pain^38^. Studies evaluating leptin levels in patients with migraine have been inconclusive, with data suggesting both low and high levels. Guldiken et al. conducted a study of 61 patients with EM and 64 controls. Fasting leptin levels were decreased in EM patients in the interictal period compared to controls^39^.

## POSSIBLE MECHANISMS INVOLVED IN THE RELATIONSHIP BETWEEN MIGRAINE AND OBESITY

Migraine and obesity may be associated in different ways, sharing genetic, biochemical, and environmental factors, as well as influencing central and peripheral mechanisms, and are associated with a high social, personal, and economic impact affecting the quality of life of individuals. The existence of a unidirectional association between migraine and obesity (i.e. obesity leading to increased migraine attacks) is clear. However, it is possible that the relationship between the two disorders could be bidirectional with migraine being a risk factor for exacerbating the development of obesity. Medications used for migraine prophylaxis such as beta-blockers, antiepileptic drugs, calcium channel blockers, and tricyclic antidepressants are known to cause weight gain. On the other hand, migraine attacks can be disabling, affecting daily life and causing a decrease in physical activity that can contribute to obesity^40^. 

Obese women have been shown to have elevated plasma levels of CGRP compared to controls. In preclinical studies, a relationship between CGRP and obesity has been demonstrated. Zucker rats are a model of genetic obesity that have a non-functional leptin receptor, which leads to hyperphagia and, consequently, obesity. These rats have elevated plasma levels of CGRP while pre-obese, although this has not been evaluated in obese mice. It appears that CGRP plays a role in the regulation of metabolism, probably linked to the CGRP receptor in the GI tract. Basal release of CGRP from meningeal afferents of the trigeminal vascular system is increased in rats with diet-induced obesity^41^.

## INSULIN RESISTANCE, METABOLIC SYNDROME AND MIGRAINE

Insulin resistance (IR) is a condition characterized by a subnormal physiological response to normal insulin concentrations, with increased amounts of insulin produced to maintain adequate intracellular glucose concentrations. Metabolic syndrome (MS) is a condition characterized by a set of metabolic abnormalities (hyperglycemia, hypertension, dyslipidemia, abdominal obesity, and pro-inflammatory state). 

In 2005 the first description of an association between migraine, IR and MS was reported, later confirmed by other studies^42^. During migraine attacks, blood glucose concentrations increase significantly and it was observed that hyperinsulinemia is associated with a 5.7-fold increased risk of migraine^43^ ^,^ ^44^. Study by Fava et al. showed that MS is significantly associated with CM (OR = 5.342, p = 0.032), and the risk of MS is significantly increased in patients with CM and medication overuse headache (OR = 12.68, p = 0.007) ^45^. 

Patients with CM have more IR than patients with EM and controls. It has also been reported that compared to healthy controls, patients with migraine with aura have a higher risk of MS (OR = 3.45; 95% CI: 1.63-7.29), which does not occur in individuals with migraine without aura^46^.

There is evidence from genetic studies that polymorphisms of the insulin receptor gene are associated with migraine^47^.

## DIABETES AND MIGRAINE

Several epidemiological studies have investigated the relationship between diabetes and migraine and it has been observed that patients with migraine do not have an increased risk of developing diabetes mellitus type 2 (DM2)^46^. On the contrary, there seems to be a lower risk of DM2 in women with migraine compared to women without a history of migraine. Fagherazzi et al. reported a univariate hazard ratio of 0.80 (95% CI: 0.67-0.96) and a linear decrease in the prevalence of active migraine during the 24 years prior to DM2 diagnosis^48^. 

Regarding type 1 diabetes (DM1), data from the *Nord-Trøndelag Health Surveys* showed that among patients with this disorder the prevalence of migraine is lower (OR = 0.47, 95% CI: 0.26-0.96) than individuals without DM1^49^. However, the biological mechanisms underlying the protective effect of diabetes on the risk of migraine attacks are unknown. 

The comorbidity between migraine and obesity, as well as the role of various dietary factors in headache attacks, have led to the investigation of different diets for migraine prevention with the ketogenic diet showing great promise in preventing migraine attacks^50^ ^,^ ^51^. 

## METABOLIC DYSFUNCTION IN IIH

**Figura 1.**
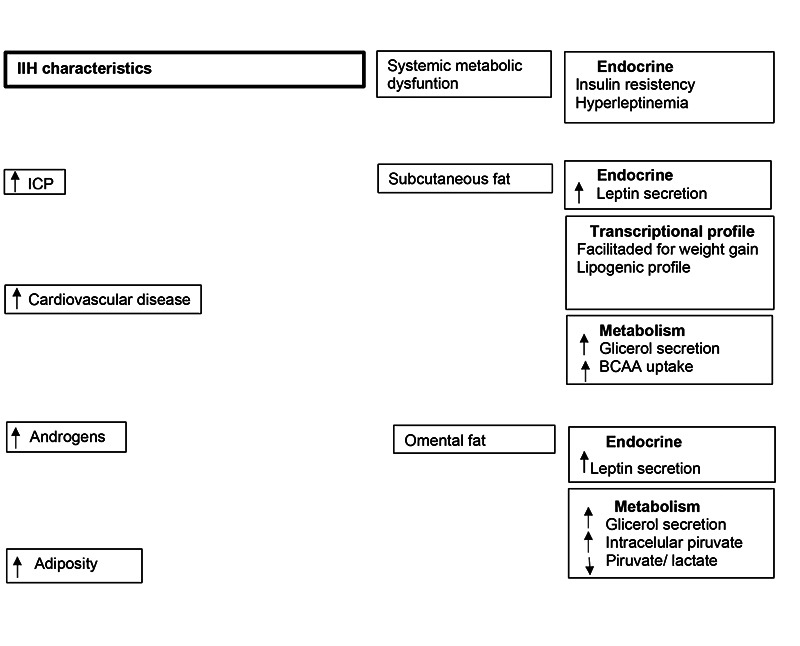
Características metabólicas dos pacientes com Hipertensão intracraniana idiopática.

Adapted from Westgate CS, Botfield HF, Alimajstorovic Z, Yiangou A, Walsh M, Smith G, et al. Systemic and adipocyte transcriptional and metabolic dysregulation in idiopathic intracranial hypertension. JCI Insight. 2021; 24;6(10):e145346. doi: 10.1172/jci.insight.145346.

IIH is a condition of systemic metabolic dysregulation with neuro-ophthalmological manifestations in which there is an excess of androgens, as in polycystic ovary syndrome. Excess androgens are linked to IR and may contribute to DM2 and cardiovascular disease. There is insulin resistance and hyperleptinemia in IIH. Figure 1 shows the metabolic characteristics of patients with IIH in relation to obese patients without IIH with the same BMI, total fat mass and total lean mass. Subcutaneous adipose tissue has comparatively increased leptin secretion and a facilitated transcription profile for energy accumulation, weight gain and lipogenesis, and has been shown to have a comparatively greater ability to uptake aromatic branched-chain amino acids, with catabolism of isoleucine and leucine. In addition, it shows increased glycerol secretion. Omental fat tissue also shows differences from that of obese non-IIH patients, such as increased leptin and glycerol secretion, increased intracellular pyruvate, and a reduced pyruvate/lactate ratio^52^.

## IMPACT OF WEIGHT LOSS ON MIGRAINE

Some studies have addressed the question of a possible influence of weight loss on migraine, particularly in CM. 

The first prospective clinical study was performed by Bond et al. to verify changes in migraine frequency and intensity six months after bariatric surgery (BS). Twenty-four patients with EM and severe obesity (BMI ≥ 35) were studied. The vast majority were women, with a mean age of 39.3. The mean BMI reduction was from 46.6 to 34.6 postoperatively. The mean number of days with headaches at postoperative evaluation decreased by almost 50% regardless of type of BS or percentage of weight loss. There was a reduction in pain intensity and severity as assessed with the *Migraine Disability Assessment Scale* (MIDAS) six months after surgery. Patients who had greater weight loss had a greater chance of a 50% or greater reduction in headache frequency and impact (p = 0.008)^53^. It must be observed that factors other than weight loss could be responsible for these results, such as high expectation in the surgical procedure. Furthermore, there was no control group. 

Another prospective study with 29 obese women with migraine who underwent BS reported that six months after surgery there was a significant reduction in the frequency and duration of migraine attacks, use of symptomatic medication, and disability caused by crises^54^. 

A retrospective trial by Gunay et al. studied 81 morbidly obese patients, with a mean age of 40, 90% of whom were women, with a mean BMI of 48, with a preoperative diagnosis of migraine undergoing Y-de-Roux gastric bypass. Migraine symptoms improved in 89% of patients within about six months after BS^55^.

## BEHAVIORAL INTERVENTIONS IN PATIENTS WITH MIGRAINE

A randomized controlled trial by Lemstra et al. evaluated the effect of a multidisciplinary program on migraine. Eighty participants (including 36 controls) were included. The program consisted of group exercises three times a week for six weeks, stress management, relaxation, dietary discussions, and massage therapy. There was a significant reduction in frequency, intensity, duration of headache, and quality of life measures in the migraine group versus the control group after six weeks. In this study, however, the participants' initial weight and its changes after the program were not specified^56^. 

Verrotti et al. reported data from 135 obese adolescents with migraine who underwent a multidisciplinary program and demonstrated that the association of specific diet, with a physical training program and behavioral therapy improved weight and migraine picture after 12 months. The frequency, intensity, and duration of attacks and the MIDAS score adapted for the pediatric population improved significantly with the established program^57^. 

A systematic review and meta-analysis studied the effect of weight loss achieved by BS or behavioral intervention on migraine frequency and severity. Ten studies (n = 473) were included in the meta-analysis and showed that weight loss leads to significant reductions in headache frequency (ES - 0.78, p < 0.0001), pain severity (ES - 1.04, p < 0.0001), disability (ES -0.68, p < 0.0001), and attack duration (ES - 0.25, p =0.017). Improvement in migraine did not correlate with either the degree of obesity at the beginning of the study or the degree of weight reduction. The effect on migraine was similar when weight reduction was achieved with BS or behavioral intervention and was comparable in adult and pediatric populations^58^. 

Therefore, weight loss, whether associated or not with diet and other behavioral interventions, was demonstrated to potentially change the evolution of migraine in individuals with severe obesity. However, the studies to date were conducted in populations with severe obesity and not in migraineurs with low-grade obesity or overweight. In this group, the impact weight loss might have on migraine and associated disability still needs evaluation. 

Ketogenic diet (KD), which is based on restricted carbohydrate intake, forcing the metabolism to obtain energy from the oxidation of fatty acids that transform into ketone bodies, can also be used by the central nervous system. KD is an established treatment for refractory pediatric epilepsy and a promising therapy for some other neurological diseases^10^ ^,^ ^59^. 

KD in the treatment of CM has recently been considered. Bongiovanni et al. published a study of 50 patients to verify symptom modification in patients with refractory CM in response to a three-month KD. Thirty-eight patients completed the study and 23 were included in the statistical analysis. Days with symptoms decreased from the mean of 30 to 7.5 (p < 0.0001). The duration of migraine episodes decreased from a mean of 24 h to 5.5 h (p < 0.0016). Patients' pain level, initially at the maximum value for 83% of participants, improved for 55% of them (p < 0.0024). The amount of medication taken in one month decreased from 30 doses (mean) to six^60^. 

Benefits of using KD were observed in the pediatric population. A literature review by Barbanti et al. on the use of KD in adolescents and children in the treatment of migraine evaluated data obtained from 150 patients investigated in case reports and prospective studies. The data suggest that KD may be an effective rapid onset option for EM and CM prophylaxis. Although the mechanism by which KD would work remains unclear, it may be that it contributes to restoring brain excitability and metabolism and to counteracting neuroinflammation in migraine^60^. However, randomized controlled studies are needed to confirm the usefulness of KD in migraine and investigate its optimal duration, and its feasibility in normal weight subjects, efficacy in the pediatric population, and association with conventional migraine prophylaxis. 

## BEHAVIORAL AND SURGICAL INTERVENTIONS IN PATIENTS WITH ICH

Sinclair et al. published the results of a prospective cohort of 25 morbidly obese women with IIH (excluding those undergoing surgery to treat IIH) who underwent a 435 Kcal/day diet for three months. The average weight loss was 15.8 kg. Improvement occurred in headache frequency and intensity, visual function, and ICP. The mean decrease in ICP was 8 cm of H2O on average^61^.

A recent systematic review which included 12 studies (n = 39 patients) of patients with IIH undergoing BS showed that the mean preoperative BMI of 47.4 ± 3.6 kg/m^2^ went to 33.7 ± 2.1 kg/m2 and 33.9 ± 11.6 kg/m^2^ at six and 12 months post-operatively, respectively. Lumbar CSF opening pressures decreased from 34.4 ± 6.9 cmH2O to 14.0 ± 3.6 cmH2O after surgery. HII symptoms improved after BS; headaches (100% preoperatively *v.* 10% postoperatively), visual complaints (62% *v.* 44%), tinnitus (56% *v*. 3%) and papilledema (62% *v.* 8%). Although the quality of the studies included in this systematic review is limited, BS seems to lead to considerable improvement in ICH as well as in headaches.^62^

In conclusion, population studies consistently show an association between obesity and headaches in general, as well as migraine specifically, both episodic and chronic. Obesity is estimated to increase the migraine risk by 40-80%, the risk increasing with increasing obesity status. The risk of the migraine-obesity association can be modified by age, being highest in those under the age of 50. The association between obesity and TTH is less robust, and may be stronger in those with CTTH compared to ETTH. IIH is a secondary headache that is significantly associated with obesity and with weight gain. Since obesity is a modifiable risk factor, it is important for physicians treating patients with headache, and particularly migraine and IIH, to be aware of the headache/migraine/obesity association.
